# miRNA-146-a, miRNA-21, miRNA-143, miRNA-29-b and miRNA-223 as Potential Biomarkers for Atopic Dermatitis

**DOI:** 10.3390/clinpract15110192

**Published:** 2025-10-23

**Authors:** Sanja Jakovljevic, Iva Barjaktarovic, Dunja Jakovljevic, Olivera Levakov, Ljuba Vujanovic

**Affiliations:** 1 Department of Dermatovenereology, Faculty of Medicine, University of Novi Sad, 21000 Novi Sad, Serbia; olivera.levakov@mf.uns.ac.rs (O.L.); ljuba.vujanovic@mf.uns.ac.rs (Lj.V.); 2 Clinic of Dermatovenereology Diseases, University Clinical Center of Vojvodina, 21000 Novi Sad, Serbia; 3 Department of General Education Subjects, Faculty of Medicine, University of Novi Sad, 21000 Novi Sad, Serbia; iva.barjaktarovic@mf.uns.ac.rs; 4 Center for Laboratory Diagnostics, University Clinical Center of Vojvodina, 21000 Novi Sad, Serbia; 5 Faculty of Pharmacy Novi Sad, 21000 Novi Sad, Serbia; dunja.jakovljevicffns@gmail.com

**Keywords:** atopic, epigenetic, biomarkers, miRNA

## Abstract

**Background/Objectives**: Recently, epigenetic mechanisms have been recognized as crucial in atopic dermatitis development. The emphasis of this research was on expanding existing knowledge about the epigenetic aspects of atopic dermatitis, as well as identifying new molecules that could serve as disease biomarkers. **Methods**: The research was conducted as a cross-sectional study examining two groups: the group with atopic dermatitis (50 patients) and the control group (50 healthy adults). The serum levels of total immunoglobulin E (IgE) and eosinophil count (Eos%) were performed in routine laboratory analyses, and the detection of microRNAs from peripheral blood was performed using RT-PCR. **Results**: Analysis of selected miRNA expressions in patients with atopic dermatitis and controls revealed that only the expression and the relative expression of miRNA-146a were statistically significantly higher in patients with atopic dermatitis than in the control group (*p* = 0.042 and *p* = 0.021, respectively). There was a weak positive correlation between miRNA-146a expression and the eosinophilia/IgE level (r = 0.22 and r = 0.25, respectively). MiRNA-21, miRNA-29b, miRNA-143 and miRNA-223 were significantly upregulated in patients with higher SCORAD (*p* < 0.001, *p* < 0.001, *p* < 0.001 and *p =* 0.015, respectively). ROC curve analysis revealed the specificity of miRNA-146a as 82% and the sensitivity as 62%. The area under the ROC curve (AUC) was 0.7, indicating its diagnostic potential. **Conclusions**: Our findings imply that miRNA-146a might serve as a biomarker of atopic dermatitis, suggesting its relevance in the development of the disease, while miRNA-21, miRNA-29b, miRNA-143 and miRNA-223 may have an impact on disease progression. Our findings provide a preliminary basis that should precede validation through larger, multicentric studies and use in diagnostics, targeted personalized treatments and monitoring of treatment efficacy in atopic dermatitis.

## 1. Introduction

Atopic dermatitis (AD) is a multifactorial, inflammatory skin disease characterized by typical clinical manifestations, including itching and a chronic-relapsing course [1,2,3,4,5]. Atopic dermatitis has a wide range of clinical presentations depending on the stage of the disease, the patient’s age and the severity of the skin lesions [2,3,4]. In the acute phase, AD lesions occur as erythematous, exudative papules and plaques, with secretion, vesicles and blisters, leaving erosions and crusts [2,4,5]. In further progression, due to inflammation, scratching and rubbing, these lesions evolve into chronic thickened, dry and lichenified plaques resulting in skin dyspigmentation [2,4,5]. Characteristic adult forms of AD include ’head-and-neck’ and ’portrait dermatitis’ affecting periorificial region, face, neck and upper trunk, hand eczema susceptible to concomitant occupational contact dermatitis, extensive skin xerosis in elderly patients or erythrodermic forms that must be distinguished from cutaneous T-cell lymphoma [2,3,4,5,6,7]. Some AD patients may experience seasonal exacerbations, accompanied by allergic rhinoconjunctivitis or allergic asthma [2]. This is consistent with the concept of ‘atopic march’ in which patients with atopic dermatitis are prone to the production of allergen-specific IgE and the development of other forms of atopy [2,8,9,10,11].

The pathogenesis of AD is complex and involves the interaction between numerous genetic factors, environmental agents and dysfunctional immunologic responses [4,5,12,13,14,15]. Genetic research has revealed that the disruption of the skin barrier plays an essential role in the development of the disease [1,2,4,5,13,14,16,17,18,19,20]. On the other side, from an immunological perspective, it is widely accepted that the basis of atopic dermatitis lies in a type 2 inflammatory response initiated by keratinocyte stimulation and releasing alarmins: TSLP, interleukin (IL)-25 and IL-33, leading to ILC2 activation, with further IL-4, IL-5 and IL-13-driven Th2 and eosinophilic inflammation in the acute phase of AD, while Th1, Th17/Th22-mediated gamma interferon (γIFN) and IL-12 are predominant in chronic lesions [1,2,4,5,16,17,18,19,20,21,22,23,24].

Recent research has focused on specific types of epigenetic mechanisms that may contribute to AD disease development [16,18,21,25,26,27,28,29,30,31,32,33,34,35,36,37,38,39,40,41,42,43,44,45,46,47,48,49,50,51]. Epigenetic modifications encompass reversible enzymatic processes of DNA methylation, histone modification and posttranscriptional non-coding RNAs’ regulatory role [36,37]. These mechanisms exert epigenetic alterations on key pathogenetic events in AD, such as the activation of the immune response, T-cell polarization and keratinocytes dysfunction [36,37]. Micro RNAs (miRNAs) are short non-coding RNAs (19–25 nucleotides) that regulate post-transcriptional gene expression [1,8,48,49,50,52]. It was demonstrated that miRNAs play a regulatory role in immune response, especially in the pathogenesis of inflammatory diseases such as atopic dermatitis [1,8,18,27,28,29,31,32,33,34,35,39,40,41,42,43,44,45,46,47,48,49,50,51,52,53,54,55,56].

With the recent introduction of novel targeted therapies in the treatment of atopic dermatitis, the importance of specific modern biomarkers and the stratification of patients have been highlighted with the ultimate goal of the implementation of personalized medicine [57,58]. MiRNAs are molecules that are stable in various biological samples, serum or other body fluids, with exceptional potential as an ideal non-invasive biomarker [1,17,59,60,61,62]. The identification of miRNAs could enhance the accuracy of early diagnosis or, especially in cases with discordance between the clinical presentation and somewhat unreliable routine laboratory parameters, to reveal distinct endotypes of AD and/or predict treatment outcome [1,17,47,59,60,61,62]. It has been shown that certain miRNAs express highly dysregulated levels in atopic patients compared to healthy controls [1,8,17,21,43,44,45,46,47,48,49,50,59,60,62]. This brings a novel perspective of their further implementation in clinical practice and the creation of more sophisticated therapeutic modalities [43,47,61,63,64]. Addressed by the need for more precise diagnostics in atopic dermatitis, we managed to identify miRNA molecules that may serve as biomarkers for diagnosis or disease severity, independent of traditional laboratory parameters.

## 2. Materials and Methods

The research was conducted as a cross-sectional study at the Clinic of Dermatovenereology, University Clinical Centre of Vojvodina, Serbia, with a recruitment period from 28 April 2022 to 6 December 2023. Two groups were formed: 1. The control group (C), consisted of 50 patients who fulfilled the required inclusion criteria (adults without any form of atopy such as atopic dermatitis, asthma, allergic rhinitis, food allergy or any other systemic disease and have not been under any immunosuppressive therapy in the last two months); 2. The experimental group (AD), consisted of 50 patients who presented either as previously or newly discovered cases of atopic dermatitis and who fulfilled the inclusion criteria (atopic dermatitis with/without any other form of atopy but without any other systemic disease and have not been under any immunosuppressive therapy in the last two months).

The exclusion criteria were as follows: patients younger than 18 years, patients who had been under any immunosuppressive therapy in the last two months, pregnant women and patients with any kind of systemic disease.

All participants signed the informed consent, and the research was approved by the Ethics Committee of the Clinical Centre of Vojvodina (protocol number #600-210).

The clinical examination of patients was performed independently by two dermatologists, and the assessment of the extent and intensity of atopic dermatitis was performed using the SCORAD index (SCORing Atopic Dermatitis). The SCORAD index was interpreted as follows: final score less than 25 presented as mild AD, between 25 and 50 as moderate AD and more than 50 as severe AD.

The serum levels of total immunoglobulin E (IgE) and eosinophil count (Eos%) were measured for each patient as routine laboratory analyses.

The detection of microRNAs was performed by a specific procedure: patients’ peripheral blood samples were collected in test tubes with a reagent for the stabilization of cell RNAs (PAXgene^®^ Blood RNA Tubes (Becton, Dickinson and Company, Eysins, Switzerland); the extraction of total microRNA was performed using a TaqMan^®^ miRNA ABC Purification Kit (Thermo Fischer, Nerum, Denmark), followed by reverse transcription of microRNAs of interest using a TaqMan^®^ MicroRNA Reverse Transcription Kit and specific primers (Thermo Fischer, Nerum, Denmark), following the manufacturer’s instructions. The exact primer and probe sequences were proprietary and not provided by the manufacturer. According to the previously published data, seven miRNAs of high importance in atopic diseases were selected. Amplification and analysis of selected microRNAs: 21, 26a, 29b, 203, 223, 143 and 146a was carried out using real-time PCR (ABI Prism 7000 Sequence Detection System, Applied Biosystems, Foster City, CA, USA) and TaqMan^®^ MicroRNA Assay reagents (Thermo Fischer, Nerum, Denmark). All reactions were carried out according to the manufacturer’s protocols.

Data analysis was performed using SPSS software, version 23.0 (Chicago, IL, USA). Quantitative data were tested for normal distribution using the Kolmogorov–Smirnov test. Normally distributed data were compared using Student’s *t*-test and nonparametric data were analyzed with the Kruskal–Wallis test. Parameters were further analyzed using binomial and multinomial logistic regression. The *p*-value of <0.05 indicated a statistically significant difference.

## 3. Results

### 3.1. Patient Dataset

Participants in this research represented adult patients of Caucasian race, older than 18 years and residents of the Autonomous Province of Vojvodina, Serbia, who were scheduled for an appointment at the Clinic of Dermatovenereological diseases. The study encompassed the total number of 100 participants, involving 71 (71%) female and 29 (29%) male participants with average age of 34.5 ± SD year (range 18–73 y). According to the order of their appointment at the clinic, patients who met the required criteria were included in either the control (C) or the experimental group (AD), counting for 50 participants per each group: the C group included 35 (70%) female and 15 (30%) male participants, while AD group included 36 (72%) female and 14 (28%) male patients. Patients in the experimental group showed disease duration (AD) of at least two weeks (average duration 7.22 years): 13 patients < 1 y, 20 patients 1–5 y, 5 patients 5–10 y; 12 patients > 10 y, among which eight patients have had AD since childhood. Regarding atopic comorbidities, the experimental group involved the patients with the following: only atopic dermatitis—22 (44%); atopic dermatitis and allergic rhinitis—16 (32%); atopic dermatitis, allergic rhinitis and asthma—7 (14%); atopic dermatitis, allergic rhinitis and food allergy—4 (8%); and atopic dermatitis and food allergy—1 (2%).

The experimental group (AD) included 6 patients with SCORAD < 25 (12%), 26 patients with SCORAD 25–50 (52%) and 18 patients with severe atopic dermatitis and SCORAD > 50 (36%). IgE levels ≤ 100 IU/mL were detected in 12 patients (24%), IgE levels 100–500 IU/mL were found in 18 patients (36%), while the remaining 20 patients expressed IgE ≥ 500 IU/mL (40%). In this group, moderate hypereosinophilia was found only in one case (values 1.5–5.0  ×  10^9^/L) (2%), while mild eosinophilia was detected in eight patients (values 0.5–1.49  ×  10^9^/L) (16%).

In the control group, IgE levels of ≤100 IU/mL were predominantly detected in 39 participants (78%). IgE levels 100–500 IU/mL were detected in 11 participants (22%), while higher levels of IgE (>500 IU/mL) were not registered. A mild absolute eosinophilia was assessed in two participants (0.51 and 0.54  ×  10^9^/L) (4%), while the absolute eosinophil count in the rest of the group was in the physiological reference interval.

A descriptive table detailing the characteristics of the 100 patients included in the study is given below (Table 1).

### 3.2. MiRNAs, Serum IgE Levels and Eosinophils in the Experimental—AD and Control Group—C

**Figure 1:**
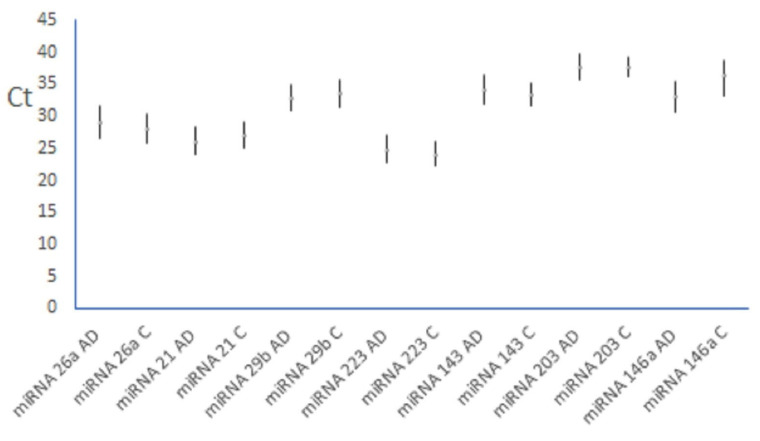
Mean Ct values for selected miRNA in both groups (mean ± 95% CI) AD—atopic dermatitis, C—controls.

The seven microRNAs (miRNA—21, 26a, 29b, 143, 146a, 203 and 223) were analyzed in the whole blood samples from 50 patients with AD and 50 controls by real-time PCR (Figure 1). The statistics were performed using SPSS software version 23.0 (Chicago, IL, USA). A *p*-value < 0.05 indicated a statistically significant difference.

The quantitative data were presented as means, with 95% confidence intervals (CI) calculated for each group (Figure 1), checked for normal distribution using the Kolmogorov—Smirnov test, and further analyzed by a two-tailed Student’s *t*-test.

The expression of analyzed miRNA in patients with AD and those from the control group were compared, and only miRNA-146a was found to be significantly upregulated in AD patients compared to controls (*p* = 0.042), while miRNA-26a, miRNA-21, miRNA-29b, miRNA-223, miRNA-143 and miRNA-203 did not show significantly different expression between groups (*p =* 0.585; *p* = 0.573; *p* = 0.665; *p* = 0.717; *p* = 0.630; *p* = 0.739, respectively). Table 2 and Figure 1 present the mean Ct values for each miRNA across the AD and control groups, with error bars indicating the 95% confidence intervals.

**Figure 2:**
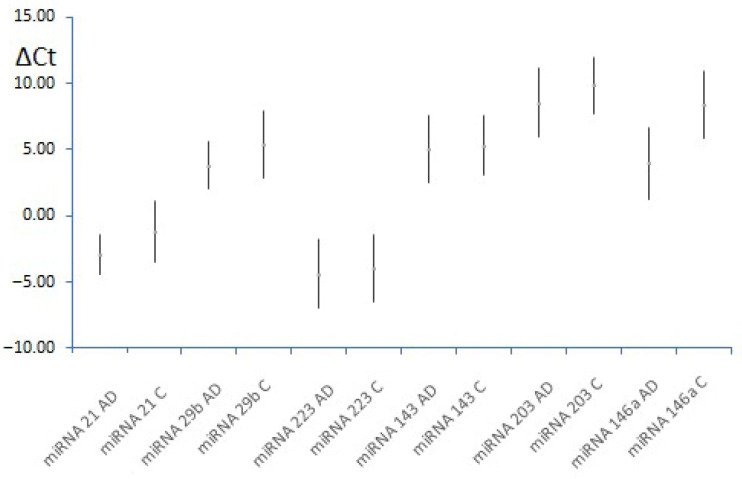
Mean ΔCt values for selected miRNAs in both groups (mean ± 95% CI). AD—atopic dermatitis, C—controls.

The relative expression of each miRNA was expressed as ΔCt, calculated as the difference in the threshold cycle (Ct) between the miRNA of interest and miRNA-26a. The relative expression, ΔCt, was calculated for each miRNA to compensate for any differences in the initial amount of miRNA in both samples that may arise due to hemolysis and cellular fractionation. miRNA-26a was used for the normalization of expression data because of the previously demonstrated similar expression among groups. Comparing all given values in both research groups revealed that, although all analyzed miRNAs were upregulated in the AD group, only the relative expression of miRNA146a was noticeably higher (*p* = 0.021) (Table 3, Figure 2).

Given the interpretation that Ct values of miRNA above 30 are classified as weak-positive, and below 30 as strong-positive, and after comparing those results with the implementation of the Kruskal–Wallis test, again, only miRNA-146a was identified with considerably higher expression in the AD group than in control group (*p* = 0.046) and therefore selected for further analysis. miRNA-146a was found to be significantly upregulated in AD patients compared to controls (*p* = 0.046), while no other miRNAs showed statistically significant differences between the groups.

**Figure 3:**
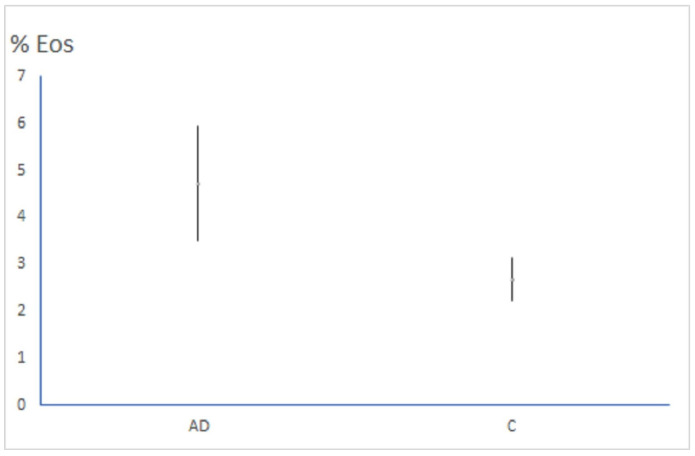
The comparison of the percentage of eosinophils between the AD and C groups (*p* = 0.012).

**Figure 4:**
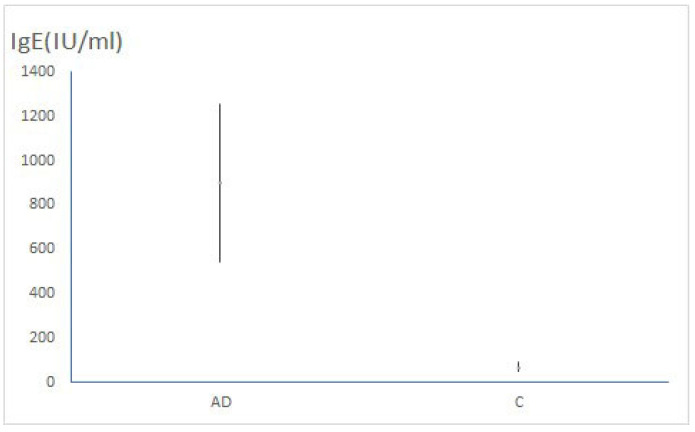
The comparison of the IgE levels between the AD and C groups (*p* < 0.001).

The level of IgE in the blood was substantially higher among patients with AD than in the control group (*p* < 0.001). Measured parameters in both research groups were additionally evaluated using binomial logistic regression, resulting in the following: the percentage of eosinophils was significantly higher in the AD group than among controls (*p* = 0.012) (Figure 3); the IgE level was markedly higher in the AD group than in the control group (*p* < 0.001) (Figure 4); and miRNA-146a was considerably more expressed in the AD group than in the control group (*p* = 0.041). After adjusting for multiple comparisons using the Holm–Bonferroni method, the results for the percentage of eosinophils, the IgE level and miRNA-146a remained significant (*p* = 0.024, *p* = 0.003 and *p* = 0.041, respectively). There was a significant, but weak positive correlation between miRNA-146a expression and eosinophilia, and between miRNA-146a expression and IgE level (r = 0.22, 95% CI: −0.06, 0.47; *p* = 0.025 and r = 0.25, 95% CI: −0.03, 0.49; *p* = 0.013, respectively).

**Figure 5:**
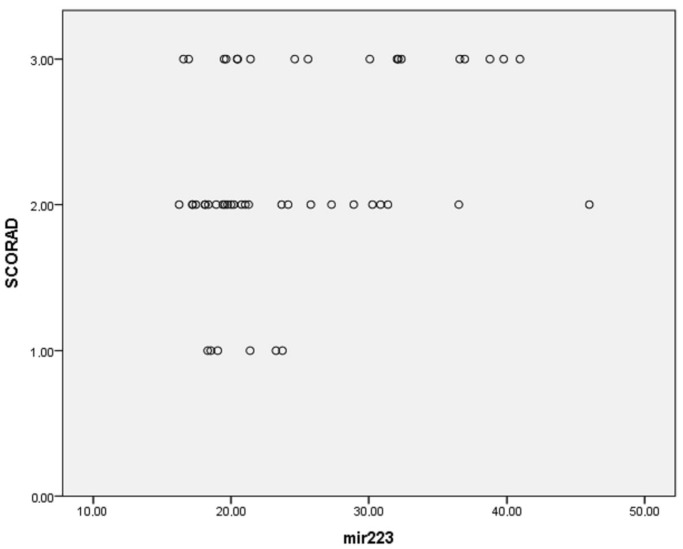
Correlation between miRNA-223 expression and disease severity (r = 0.342, *p* = 0.015).

Assessing the correlation between measured parameters in the blood (IgE and Eos) and SCORAD resulted in the following: the eosinophil percentage was in significant correlation with SCORAD (r = 0.36, 95% CI: 0.09, 0.58; *p* < 0.001), as was the IgE level (r = 0.32, 95% CI: 0.05, 0.55; *p* = 0.025). There was also a moderate positive correlation between miRNA-223 expression and SCORAD (r= 0.342, 95% CI: 0.07, 0.57, *p* = 0.015) as well as between miRNA-146a expression and SCORAD (r= 0.191, 95% CI: −0.09, 0.45, *p* = 0.05). (Figure 5).

**Figure 6:**
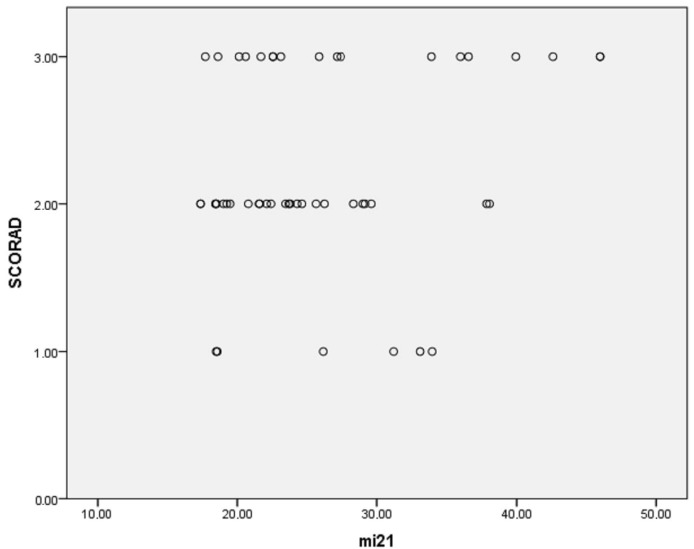
Association between miRNA-21 expression and disease severity (*p* < 0.001).

**Figure 7:**
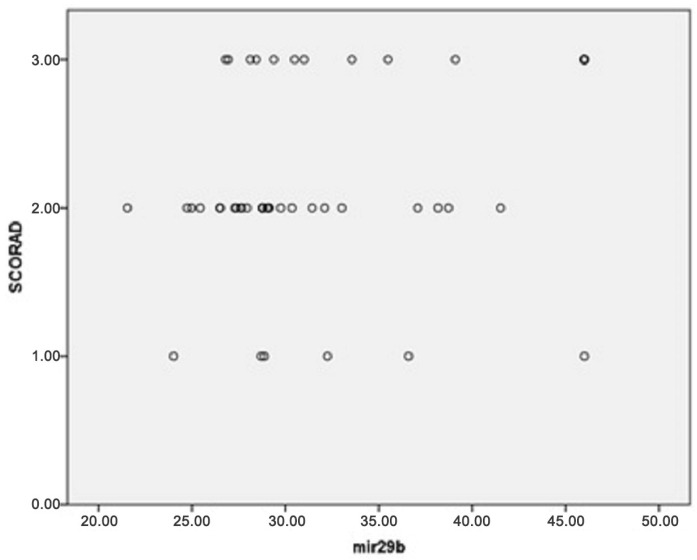
Association between miRNA-29b expression and disease severity (*p* < 0.001).

**Figure 8:**
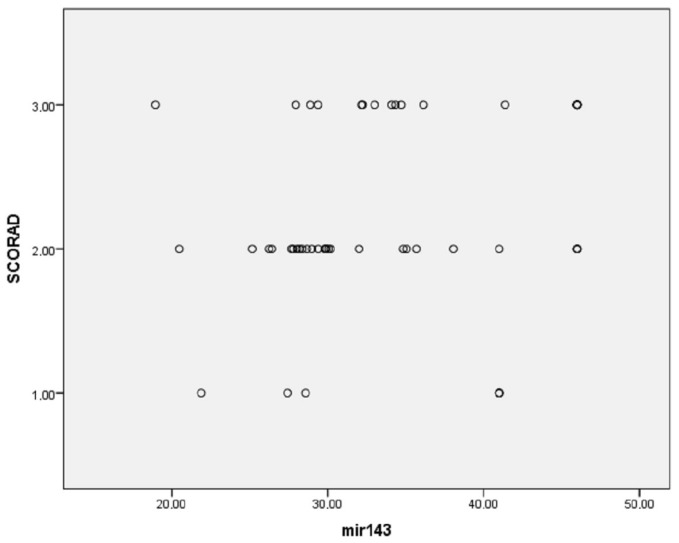
Association between miRNA-143 expression and disease severity (*p* < 0.001).

Additionally, multinomial logistic regression was performed to adjust for confounding factors (age, gender, disease duration, presence of allergic rhinitis (AR), asthma and food allergy), resulting in the following findings: the percentage of eosinophils was considerably higher in patients with higher SCORAD (*p* < 0.001); miRNA-21, miRNA-29b and miRNA-143 were significantly more expressed in patients with higher SCORAD (*p* < 0.001 for all three miRNAs; Figure 6, Figure 7 and Figure 8, respectively). The adjusted *p*-values remained statistically significant after applying the Holm–Bonferroni correction (*p* = 0.004 for all three miRNAs).

**Figure 9:**
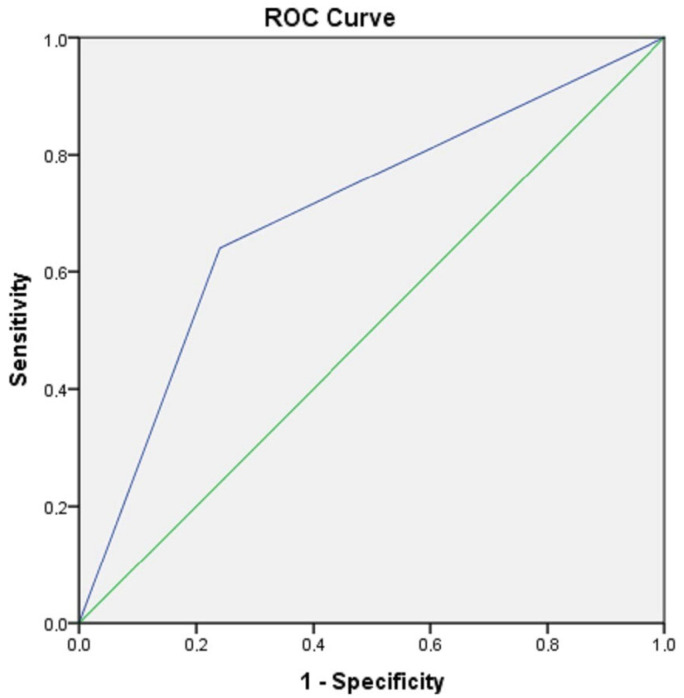
ROC curve analysis revealed that miRNA-146a may present a valuable biomarker for AD (AUC = 0.7. *p* = 0.04). blue—ROC curve, green—reference line.

MiRNA-146a’s diagnostic potential was evaluated based on ROC curve analysis (receiver operating characteristic curve). The area under the ROC curve (AUC) was used to establish the diagnostic applicability of miRNA-146a in AD patients. The optimal diagnostical result was estimated by comparing given values to borderline values with the highest Youden’s index (sensitivity +1, specificity −1). ROC curve analysis revealed that the specificity and sensitivity of miRNA-146a were 82% and 62%, respectively. The area under the ROC curve (AUC) was 0.7, which confirms that miRNA-146a has diagnostic potential in AD (Figure 9). Therefore, miRNA-146a may be an appropriate biomarker of AD.

## 4. Discussion

### 4.1. miRNAs in Atopic Dermatitis

MiRNAs are small molecules consisting of 19–25 nucleotides that belong to non-coding RNAs [1,8,48,49,50,52]. Their exceptional role in the regulation of posttranscriptional gene expression has been recognized as an essential factor in immunity and pathogenesis of various inflammatory diseases, such as atopic dermatitis [1,8,16,21,43,46,47,48,53,54,55,56,65,66].

According to available data, several miRNA molecules might be involved in epidermal regulation—increased levels of miRNA-203 and miRNA-184 are associated with dysregulated expression of filaggrin, involucrin and loricrin, while miRNA-194-5p is involved in the regulation of gene expression of structural proteins—LOR, KRT4, FLG2 and heparan sulfate—HS3ST2 [25,36,38]. Additionally, dysregulated levels detected for miRNA-10a-5p and miRNA-26a-5p, which are involved in the regulation of HAS3 (hyaluronan synthase 3), leads to inadequate keratinocyte differentiation and migration [21,36,37,43,44,47]. Epidermal dysfunction in AD may also be substantially due to miRNA-29b promoting keratinocyte apoptosis via the inhibition of Bcl2L2 (Bcl-2-like protein 2) [30].

In AD, certain miRNA molecules exhibit potentially immunosuppressive activity, such as miRNA-124, miRNA-1294, miRNA-205 and miRNA-146a via regulating crucial parts of the NF-κB pathway in keratinocytes and different immune cells, or miRNA-143 via targeting IL-13Rα1 in keratinocytes silencing IL-13-mediated decreased expression of filaggrin, involucrin and loricrin [21,27,28,29,37,39,40,43,46,47]. Concerning proinflammatory miRNAs, miRNA-144 may have been of high importance in AD by regulation of hBD-2 and SERPINB4—NF-κB activators, while decreased levels of let-7a-5p in AD relate to the overexpression of CCR7 on T-cells and DCs in atopic patients [37,43]. In the context of T-cell differentiation, several miRNAs are particularly important. For instance, miRNA-155 expression was found to positively correlate with the percentage of Th17 cells, levels of IL-17 and RORγ (retinoic acid-related orphan receptor), but negatively with SOCS1 (signaling inhibitor suppressor of cytokine signaling-1) and CTLA-4 (cytotoxic T lymphocyte-associated antigen 4) [31,32,33,37]. Additionally, miRNA-151a may have an inhibitory effect on IL12Rβ2/JAK/STAT, which is included in Th1 differentiation, while miRNA-191 affects Treg cell activation by targeting SATB1 (AT-rich sequence-binding protein 1) [34,37,46]. Lastly, miRNA-302e and miRNA-135 showed antiallergic effects via inhibiting NF-κB and GATA-3, respectively, resulting in the suppression of Th2 differentiation and cytokine production [42]. Similarly, the suppressive effects that AD exhibits are also related to miRNA-193, which negatively regulates HMGB1 (high mobility group box 1) and NF-κB pathways, resulting in keratinocyte proliferation, epidermal restoration and subsided inflammation. It was shown that miRNA-193 overexpression was promoted via the upregulation of transcription factor Sp1 (specificity protein 1) [67]. In terms of the epigenetic profile of lesional AD, the overexpression of miRNA-223 in AD lesions is associated with T-cell regulation and subsequent inflammation [21,43,47]. On the other hand, miRNA-21 presented a protective role through the inhibition of the p38 pathway with subsequent suppression of mast cell degranulation, skin inflammation and scratching behavior in an animal model [42]. Concerning atopic comorbidities, miRNA-4497 has been recently highlighted as a consistently downregulated miRNA in sera of pediatric patients with atopic diseases. Experimentally, it was shown that the transfection of miRNA-4497 ameliorated allergic inflammation in mast cells, which was reflected in lessened IL-4, macrophage-derived chemokines and methacholine secretion [68].

Regarding epigenetic regulation and microbiome in AD pathogenesis, highly upregulated miRNA-939 was found to promote the expression of several matrix metalloproteinase genes (MMP1, MMP3 and MMP9) and intercellular adhesion molecule 1 (ICAM1) in the keratinocytes of AD lesions. Accordingly, in vivo studies underpinned that the overexpression of miRNA-939 supported colonization of St. aureus and eczema deterioration via enhanced expression of MMPs, implying that miRNA-939 might be a promising therapeutic target [69].

Everything previously mentioned highlights the significant role of miRNAs in modifying allergic inflammation in atopic diseases, through regulation of the production of proinflammatory and anti-inflammatory molecules and cell biology, resulting in an impaired epidermal barrier, disruption of immune homeostasis and the eventual development of atopic dermatitis.

### 4.2. miRNA-146a, miRNA-21, miRNA-29b, miRNA-143, miRNA-223, miRNA-203 and miRNA-26a

It was established that certain microRNAs were present in patients with atopy, or that their levels significantly deviated from the values obtained in subjects who did not have any form of atopic disease [1,8,21,47,48,50]. For our study, we selected seven miRNAs for further investigation based on previous publications [1,8,17,21,37,43,44,45,46,47,48,49,50,53,54,55]. Our results revealed that only miRNA-146a’s relative expression was statistically significantly higher in AD in comparison to the C group values (*p* = 0.021). After performing ROC curve analysis, the diagnostic potential of miRNA-146a was validated (Figure 9). Recent studies on atopic dermatitis established the overexpression of miRNA-146a in the skin lesions and blood of AD patients [8,26,28,29,43,44,46,47,49,55,70,71]. Our results support available data that miRNA-146a might serve as a promising diagnostic biomarker for atopic dermatitis (*p* = 0.021, AUC = 0.7), presuming its prominent role in the pathogenesis of the disease.

Experimental studies revealed potential mechanisms through which miRNA-146a may regulate innate and adaptive immunity, antibody production, inflammation, haemopoiesis, differentiation and the proliferation of keratinocytes [28,29,43,46,55,72,73,74,75]. Thus, miRNA-146a is involved in oncogenesis, the pathogenesis of autoimmune disease and allergic response [75]. The studies on miRNA-146a expression revealed its clinical implications, serving as a valuable diagnostic and prognostic biomarker, while different models demonstrated molecular mechanisms underlying the development of various diseases [75]. It was found that miRNA-146a controls the suppression of TNF-mediated osteoclastogenesis in rheumatoid arthritis (RA), while the decreased expression of miRNA-146a due to the rs2431697 variant in the miRNA-146a gene in systemic lupus erythematosus (SLE) can lead to altered IFN signaling and progression of the disease [75]. Conversely, one study detected a protective role of single-nucleotide polymorphism (rs2910164) in the gene encoding precursor of miRNA-146a in the early onset of psoriasis [74]. The authors proposed that miRNA-146a overexpression resulted in ameliorating IL-17-mediated inflammation, decreased neutrophil influx and regulation of keratinocyte hyperproliferation in psoriasis [74]. While EGFR has been verified target for miRNA-146a, another study identified FERMT1 as a psoriasis-related miRNA-146a target gene involved in keratinocyte proliferation [72,74].

About miRNA-146a’s regulatory role in cell biology and differentiation, available data indicates that Treg cells exert the capacity of suppressing CD4 + CD25- T cells by the change in their intracellular miRNA-146a levels, while Th2-differentitation can be inhibited via miRNA146a targeting SERPINB2 (serpin family B member 2) [29,72,76,77]. Additionally, miRNA-146a may be responsible for B cell class switching and the increased production of IgE antibodies via upregulation of 14-3-3σ expression in the asthma animal model [73]. Research conducted on miRNA-146a-deficient mice suggested the existence of a link between the serum IgE level and the expression of miRNA-146a, while patients’ serum miRNA-146a levels were independently associated with IgE levels in AD, without correlations to the severity of AD [70]. In our research, a weak correlation was observed between miRNA-146a and IgE levels (r = 0.25, *p* = 0.013) in the AD cohort, while its correlation with SCORAD was moderate (r = 0.191, *p* = 0.05). Conversely, in a study on allergic conjunctivitis, the authors indicated the inverse correlation between miRNA-146a and serum IgE levels [76].

MiRNA-146a inhibits the production of proinflammatory molecules ubiquitin D, CCL5 and CCL8, and suppresses their expression in epidermal keratinocytes, but also adjusts the activity of B-cells, T-lymphocytes, mast cells, monocytes and dendritic cells [17,43,44,47,49,78,79]. Both tissue culture and animal models demonstrated that miRNA-146a is involved in the NF-κB pathway, targeting the upstream mediators IRAK1 (IL-1 receptor-associated kinase 1), CARD10 (caspase recruitment domain containing protein 10) and TRAF6 (tumor necrosis factor receptor-associated factor 6), ameliorating chronic inflammation [17,21,28,37,45,46,49]. The downregulation of adapter proteins IRAK1 and TRAF6 causes reduced activation of NLRP3, inhibition of TLR-signaling and in the final step leads to the significant reduction in IL-6, IL-18, IL1β and αTNF release [75,80].

To our knowledge, the miRNA profile of eosinophils does not express significant miRNA-146a levels nor was the correlation with blood eosinophil number detected [81,82,83,84,85], as it was demonstrated for miRNA-21 and miRNA-223 to be involved in eosinophil biology: lower expression of miRNA-223 correlates to increased levels of eosinophil progenitors and eosinophil differentiation via targeting IGF1R [21,43,47,86], while miRNA-21 also plays a role in IL-5-mediated eosinophil differentiation via direct regulation on eosinophil progenitors [42,87]. In our study, a weak but significant positive association between the miRNA-146a expression and eosinophils was detected (r = 0.22; *p* = 0.025), suggesting the potential indirect biological relation between upregulated miRNA-146a and eosinophilia. Several allergic models demonstrated miRNA-146a’s direct impact on ILC2 cells’ function and proliferation via IL-33/ST2 signaling through the negative regulation of IRAK1 and TRAF6, leading to a decreased release of IL-5 and IL-13 and subsequent amelioration of eosinophil inflammation [88,89]. Similarly to previously, in the allergic rhinitis model (AR) and in β-lactoglobulin-induced food allergy, miRNA-146a showed anti-inflammatory potential by inhibiting the TLR4/TRAF6/NF-κB pathway [90,91]. For instance, in the AR study model, administration of miRNA-146a into the nostril resulted in decreased levels of OVA-specific IgE, LTC4, PGD2, ECP, IL-4, IL-5 and IL-13, as well as a decreased number of mast cells and reduced infiltration of eosinophils, lymphocytes and neutrophils, while converting Th1/Th2 imbalance and preventing an IgE-mediated response [90]. Another experiment proposed that miRNA-146a inhibitors might lessen asthmatic inflammation via upregulation of the TLR2-signaling pathway in monocytes [92]. Lastly, in animal models of allergic conjunctivitis, the pollen-induced downregulation of miRNA-146a was followed by the enhanced expression of TSLP/TSLPR/OX40L/CD11C molecules, while the other investigation revealed that the overexpression of miRNA-146a induced the upregulation of FOXP3, one of the essential transcription factors in promoting Treg cells, the further suppression of inflammation through targeting HIPK3 (homeodomain-interacting protein kinase 3) and reduced phosphorylation of STAT3 [93,94].

It is noteworthy to mention the latest findings on type-2 inflammation in allergic diseases as they relate to the unique miRNA profile [95,96]. For instance, in an allergic asthma model due to exposure to house dust mites or in another one, with a mouse infected with Heligmosomoides polygyrus, miRNA-15a-5p, miRNA-20b-5p, miRNA146a-5p, miRNA-155-5p and miRNA-200c-3p successfully differentiate Th2 cells (IL-4+) profile [96].

The need for reliable and accurate biomarkers of disease is an imperative of modern medicine. Regarding allergic diseases, upregulated levels of miRNA-146a in asthma patients may be a part of the unique profile of miRNAs serving as a biomarker characteristic for asthma, while the miRNA profile in allergic rhinitis is quite specific and differentially expressed than in asthma patients [1,8,97,98,99]. On the other hand, miRNA-146a was specifically proven as significantly involved in the effects of allergen-specific immunotherapy in children with allergic rhinitis, highlighting this miRNA as a potential target for future therapies [1,100]. Additionally, after examining miRNA expression in tissue biopsies of eosinophilic esophagitis, as well as patients’ plasma, miRNA-146a was identified as a valuable biomarker in monitoring the effectiveness of glucocorticoid treatment [49,101]. With regard to skin diseases, upregulated levels of miRNA-146a were validated as an accurate indicator of active psoriasis [43,46,72,74,102,103]. Other skin diseases, in which miRNA-146a might be involved in their development, are oral lichen planus, vitiligo, hidradenitis suppurativa and cutaneous lupus erythematosus [43,46,53,54,56].

Evaluating the relationship between laboratory findings, clinical severity of AD and miRNAs, it has been shown that miRNA-21, miRNA-29b, miRNA-143 and miRNA-223 were significantly more expressed in patients with higher SCORAD (*p* < 0.001, *p* < 0.001, *p* < 0.001 and *p* = 0.015, respectively). MiRNA-21 is mostly studied in oncology, cardiovascular diseases, inflammatory skin diseases and asthma [43,46,104,105,106,107]. Its role in the stimulation of keratinocyte proliferation, apoptosis inhibition, angiogenesis, activation of T-cells regardless of the subtype, and relation to the IL-22 axis in maintaining inflammation in psoriatic lesions, was recognized through different research [43,46,56,107]. The overexpression of miRNA-21-3p is associated with reduced expression of caspase-14, which participates in keratinocyte differentiation and barrier formation [38]. Caspase-14 is an enzyme that cleaves filaggrin monomers to pyrrolidone carboxylic acid (PCA) in corneocytes, generating skin’s natural hydration. It was shown that PCA and caspase-14 levels are decreased in inflammatory AD lesions, manifesting as significant epidermal impairments and clinical severity of eczema [108]. In the allergic murine model, the upregulation of miRNA-21 promoted Th2 polarization, targeting IL-12p35 [8,109]. Overexpressed miRNA-21 was found in the skin lesions of allergic contact dermatitis in humans and mice, skin lesions of atopic eczema and mucosal biopsies of eosinophilic esophagitis [45,47,48,55,101,110]. However, there is currently limited data on the relationship between atopic dermatitis and miRNA-21 expression. There are a few recent creditable studies to mention. The study on the potential protective miRNAs in mother’s milk in infants younger than six months emphasized miRNA-375 as a significant factor in reducing atopy risk, while excluding the same for miRNA-21 and miRNA-146a [111]. Assessing the dysregulation of miRNAs’ expression, both in PBMCs and plasma, after six weeks of application of topical corticosteroids (TCS) in infants with AD, the research data depicted significantly altered levels of miRNA-143 in PBMCs, as for miRNA-146a and miRNA-21 in plasma, bringing further discussion on effects of TCS and epigenetic aspects of pediatric AD [111]. According to the investigation conducted to establish the association between AD in infants and particular miRNAs in breast milk, the authors hypothesized that exposure to miRNA-21 in predisposed infants may affect their immune system and result in the early development of AD [1,112]. We did not identify miRNA-21 as statistically significantly dysregulated in AD patients (*p* = 0.573), but after applying multinomial logistic regression, we found that the miRNA-21 was significantly more expressed in patients with higher SCORAD (*p* < 0.001), indicating the potential of miRNA-21 as a severity biomarker and its role in disease progression. Current results are in contrast to a recent study that identified overexpressed miRNA-21 in skin samples and the sera of patients with AD and psoriasis, with no observed correlation to the severity of AD [107].

A few studies have demonstrated that miRNA-29b was overexpressed in the lesional skin of AD patients [17,28,30,44,47,55]. Also, research showed higher miRNA-29b expression in the sera of AD patients and its positive correlation with SCORAD, suggesting that miRNA-29b may be a potential biomarker of AD [30]. Our results confirmed this finding, as miRNA-29b was significantly expressed significantly more in patients with a higher SCORAD in our study (*p* < 0.001), but not significantly more expressed in the sera of AD patients compared with healthy controls (*p* = 0.665). Therefore, we can confidently imply that miRNA-29b serves as a reliable severity biomarker in AD patients and may be responsible for the progression of atopic eczema. Excessive keratinocyte apoptosis is one of the leading features in AD, resulting in spongiosis and clinical manifestation of eczema [18,113]. Previous studies identified the γIFN-induced upregulation of Fas that culminates in keratinocytes apoptosis along with the altered expression of genes related to apoptosis (NOD2, DUSP1 and ADM) [18,113]. In addition, research on the pivotal role of NLRP10 in control of the epidermal differentiation process accentuated the capacity of NLRP10 to prevent caspase-8 intervention to the death-inducing signaling complex (DISC), as well as NLRP10 competence to stabilize p63 and physiological epidermal differentiation, maintaining barrier integrity [114]. An experimental part of the previously mentioned study proved that miRNA-29b overexpression was involved in the epidermal barrier dysfunction in AD, based on mediating γIFN-induced keratinocyte apoptosis via negative regulation of BcL2L2, an anti-apoptotic protein, contributing to the development of AD [30]. Therefore, the authors proposed the miRNA–29b/BcL2L axis as promising therapeutic target [30]. However, miRNA-29b was not detected among IL-4-modified miRNAs related to keratinocyte apoptosis, as delineated in a study for several other miRNAs [18].

Given the fact that miRNA-143 is one of the most expressed miRNAs in the skin, it was quite expected that this miRNA played a crucial role in the pathogenesis of cutaneous diseases [47]. Exploring the atopic lesional skin, scientists detected the downregulation of miRNA-143 [31]. Further experimental studies revealed that miRNA-143 mediated Th2-driven inflammation by targeting IL-13Rα1 in keratinocytes, resulting in IL-13 suppression and, presumably, epidermal barrier restoration via the regulation of filaggrin, loricrin and involucrin [17,40,44,47,48]. It was also shown that miRNA-143, via targeting IL-13Rα1, suppressed mast cell activation and allergic response, as well as overall IL-13-mediated inflammation in AD [40,42,46,115]. The authors emphasized the tremendous therapeutic potential of miRNA-143 in AD due to its effects on the IL-13 pathway [21,43,46,116,117]. To evaluate the capacity of miRNA-143 as a valid biomarker for skin inflammatory diseases, a few studies proposed that the upregulated miRNA-143 levels in PBMCs could have a diagnostic purpose both in infant AD and psoriasis patients, while in the cases of psoriasis, miRNA-143 may serve as a severity biomarker and in monitoring the treatment response as well [43,59,111,118]. The reliability of miRNA-143 expression as a diagnostic biomarker for adult AD is questionable, as the results in our AD cohort were found to be statistically insignificant (*p* = 0.630). However, our results indicate that miRNA-143 might be a valuable severity biomarker as its overexpression was detected in patients with higher SCORAD (*p* < 0.001).

Investigations on allergic contact dermatitis identified the upregulated miRNA-223 levels in human skin lesions, proposing its involvement in T cell activation and consequent cutaneous inflammation [47,56]. Apart from that, the overexpression of miRNA-223 found in the skin changes in atopic eczema [31]. A recent study ascertained that the correlation between downregulated miRNA-223 and profuse of neutrophil extracellular traps (NETs) contributes to cutaneous AD inflammation [119]. Another group of authors examined the association between miRNA-223 expression and Treg cells following prenatal tobacco exposure and its effects on children in their first three years of life. It was demonstrated that the upregulation of miRNA-223 was associated with a decreased Treg cells number in both cord and maternal blood, accompanied by a higher risk of AD development [47]. In addition, it was established that plasma levels of miRNA-223 in severe AD patients (EASI > 20) were significantly increased, correlating positively with serum TARC levels as well, pointing out the association with Th2 cell activation [47,62]. Additionally, another study conducted on AD patients and an OVA-induced AD mice model revealed similar findings: miRNA-223 was significantly elevated in the blood from AD patients and the mice model, with clinical potential to become a valuable diagnostic biomarker, while miRNA-223 overexpression was also associated with more extensive eczema lesions in the mice model [47,120]. Although miRNA-223 has been reported as upregulated in several studies of AD, our study found no significant difference in expression between AD patients and controls (*p* = 0.717). This discrepancy may be due to differences in patient populations, experimental methods or disease severity across studies. There was also no correlation between miRNA-223 levels and IgE (*p* = 0.67) and miRNA-223 levels and eosinophil count (*p* = 0.707) in our cohort. However, a moderate positive correlation was observed between miRNA-223 levels and SCORAD (r = 0.342, *p* = 0.015).

Among other miRNAs examined in our research, we would like to refer to miRNA-203 and miRNA-26a. MiRNA-203 is highly specific for skin, almost exclusively found upregulated in keratinocytes in psoriatic lesions but also in AD [55,71]. Downregulated miRNA-203 was also identified in early mycosis fungoides, distinguishing it from AD [53,121]. It was shown that miRNA-203 is involved in inflammation, keratinocyte differentiation and angiogenesis, by targeting SOCS-3, the LXRα/PPARγ axis, regulating αTNF, IL-8 and IL-24, and also in JAK2/STAT3 pathway activation, promoting further VEGF secretion [43,46,55,71,103]. MiRNA-203 stimulates epidermal differentiation through the transcription factor p63 [36,41]. Altered expression levels of filaggrin, involucrin and loricrin correlated to increased levels of miRNA-203 [38]. The overexpression of miRNA-203 is associated with the calcium-dependent activation of AP-1 (activating protein 1). It was experimentally shown that the suppression of the C/EBPα/miR-203 (CCAAT/enhancer binding protein α) axis leads to reduced expression of involucrin through the activation of DNp63α [38]. The overexpression of miRNA-203 and oleic acid found in sebum leads to the accelerated maturation of keratinocytes, while miRNA-203 also participates in the sebaceous lipogenesis of linoleic acid and ciglitazone [38]. Assessing the clinical potential of miRNA-203 in pediatric AD, scientists found that miRNA-203 serum levels were significantly elevated and in a positive correlation with the increase in serum TNFRI and TNFRII, whilst they detected the decreased miRNA-203 expression in urine and the inverse relation to serum IgE levels, suggesting miRNA-203 as an appropriate disease biomarker [17,43,47,60]. However, miRNA-203 expression was not significantly altered in our study group compared to healthy controls (*p* = 0.739), nor was the association with the measured blood parameters (Eos and IgE) or SCORAD established (*p* = 0.79, 0.454 and 0.784, respectively). The observed discrepancy in our results and available data might be due to the age-dependent existence of distinct miRNA profiles among the AD patients.

The clinical importance of miRNA-26 was established in tumorigenesis and metabolic diseases [122,123]. It was found that lower levels of miRNA-26b contribute to enhancing inflammation via cyclooxygenase-2, but miRNA-26a/b also exhibited tumor-suppressive features and had an anti-inflammatory function in type-2 diseases such as atopic dermatitis and asthma [53,96]. Bioinformatic analysis, particularly in atopic dermatitis, identified the essential genetic targets of miRNA-26a, such as hyaluronan synthase 3 (HAS3) and also CHAC1, while in asthma, miRNA-26a influenced SMAD2,3 and TGF-β-related signaling pathways [1,8,37,44,124,125,126]. Hyaluronan (HA) is an extracellular matrix glycosaminoglycan synthesized by hyaluronan synthases (HAS1, HAS2 and HAS3) in keratinocytes [127]. An experimental model detected HAS1 as the main enzyme in the physiological process of hyaluronan synthesis during normal keratinocyte differentiation, while enhanced HA levels were found in AD lesions due to the upregulation of HAS3 [127]. Additionally, the overexpression of HAS2 and HAS3 can be attributed to IL-4, IL-13 and γIFN in pathological skin conditions [127]. CHAC1 is a γ-glutamyl cyclotransferase that degrades glutathione, presenting as a paramount regulator of oxidative stress. CHAC1 expression is related to several transcription factors such as p63, which is involved in the repression of keratinocyte differentiation in AD while influenced by IL-4/IL-13 pathways [128]. The demonstration of epigenetic modulations following the application of AD IgG induced infant intrathymic nonatopic T cells to acquire IL-17/IL-22 profile or CD4+CLA+ profile, presenting downregulated miRNA-26a-5p as a certain co-regulator in AD inflammation [129,130]. Multi-omics-based identification of hub genes in AD and their miRNAs associations evinced that miRNA-26a-5p paired with hub genes MCM7 and ESR1, as well as miRNA-26b-5p and miRNA-26a-1-3p with EEF1A1 and TPF53, respectively [131]. MCM7 expression is associated with skin proliferative disorders, while ESR1 and TP53 are among genes linked to integrins, endothelins and IL-3, IL-5 and IL-8 pathways. The authors also revealed that the link between MCM7 and TNFα/NF-κB inflammation may be crucial in AD [131]. The decreased expression of miRNA-26 was identified in several studies regarding inflammatory skin lesions [30,31,53,56]. Furthermore, downregulated miRNA-26a/b levels were detected in urine in the children with AD, while the other study found upregulated plasma levels of miRNA-26a-1-3p in pediatric AD [25,43,45,60]. Our study did not reveal significantly differentially expressed miRNA-26a levels in the AD group compared to controls (*p* = 0.585).

Having meticulously explored the published data, we noticed that the scientific literature on this topic consists of numerous reviews and heterogenous results from a few clinical investigations of patients with AD that mostly include the pediatric population, small cohorts, screening of skin lesions or other inflammatory conditions such as psoriasis and asthma. We devoted exclusive attention to adult AD patients and brought novel results of the simultaneous assessment of several heretofore acknowledged miRNAs, as well as their potential clinical significance. Inevitable discrepancies among results from previous publications and this research may be due to different miRNAs’ expression in various tissue types, cells or body compartments, severity and clinical presentation of AD phenotypes, demographic parameters or application of particular eligibility criteria. The main signature of our study is the exploratory nature of the research aimed at better understanding of epigenetic aspects of atopic dermatitis and miRNAs’ role in disease development and progression. This approach of the investigation and the evaluation of specific miRNAs expand upon prior, in some measures scant, evidence in this evolving domain of science. Certainly, our results provide a rationale for further critical in-depth analysis of subtle nuances of miRNAs’ expression between subgroups of patients with atopic dermatitis that should be validated through larger cohort multicentric studies.

### 4.3. Therapeutic Potential of miRNAs

Apart from the role of miRNAs as biomarkers in early diagnosis or disease prognosis, their therapeutic potential has been recognized widely [1,21,43,47,63,64]. The research on this subject across diverse diseases is developing rapidly. The findings from such studies open new possibilities for the treatment of various disorders, given that epigenetic deviations are relatively reversible and susceptible to modification [1,21,43,47,63,64].

There are two main approaches to miRNAs administration, either by substitution or inhibition of their activity [43]. The replacement of downregulated miRNAs may be reached via the application of miRNAs mimics (agomiRs), while conversely, miRNA antagonists (antagomiRs) may be potent therapeutic agents in cases of aberrantly upregulated miRNAs across different diseases [43,63,64]. Additionally, the concept of an ‘organ-specific’ rather than ‘disease-specific’ miRNA profile proposed by one group of authors through their comprehensive review on the usefulness of antagomiRs in lung diseases should be kept in mind. [63] They pointed out that miRNA-21 was identified as most frequently dysregulated and involved in the underlying pathogenetic mechanisms of lung diseases [63]. For instance, with regard to allergic conditions, several studies demonstrated successful inflammatory modulation via the administration of anti-miR-21, resulting in reduced PI3K activity, restored HDAC2 (Histone deacetylase 2) and suppressed airway hyper-responsiveness, or reduced eosinophil count, IL-5 and IL-13 levels, depending on the study [63]. According to these authors, the most critical point may be the proper selection of essential target miRNAs that will yield the best outcome [63]. MiRNAs serve as master regulators of different biological processes, expressed in different tissues, having a wide impact on cellular mechanisms and immune modulation [1,21,43,47,63,64]. Thus, the implementation of miRNA agonists or antagonists should be wisely optimized due to their cell-specific or organ-specific expression [21].

Notably, some miRNAs are constitutionally found in the skin, among which are miRNA-21, miRNA-143 and miRNA-203, implying their exclusive influence on both the maintenance of skin homeostasis and pathogenetic contribution to disease development [47]. Concerning skin diseases, miRNA-146a, miRNA-155 and miRNA-223 expressed altered levels in the skin of AD and allergic contact dermatitis, indicating an overlap in pathogenetic processes between these conditions [47]. Studies conducted on psoriasis similarly showed upregulated miRNA-146a and miRNA-155 levels in psoriatic and atopic lesions [43,46,55,56,72,103]. Other skin diseases in which dysregulated miRNA-146a might be involved in disease development are oral lichen planus, vitiligo, hidradenitis suppurativa and cutaneous lupus erythematosus [43,46,53]. Additionally, miRNA-21 was found upregulated in the lesions of hidradenitis suppurativa and psoriasis, while one study showed that targeting miRNA-21 may offer a promising approach to treating psoriasis [43,46,132]. A few experimental models demonstrated significant amelioration of psoriasis resulting in less hyperplasia after administration of anti-miRNA-21, similarly to successful challenge with anti-miRNA-31 or anti-miRNA-210, which led to the alleviation of inflammation, epidermal changes and severity of psoriasis [43,132]. Furthermore, the application of miRNA-146, miRNA-145-5p, or miRNA-310 mimics also efficiently reduced inflammation from psoriasis [43,132]. Several studies investigated the role of miRNA-29 in collagen synthesis and fibrosis. MiRNA-29 levels were dysregulated in scars, keloids and in patients with scleroderma [43,45]. Given the fact that miRNA-29 has an antifibrotic role, it was proposed that agonist-miRNA-29 may serve as a novel treatment option in cutaneous fibrotic disorders and excessive scarring [43]. In the field of oncology, miRNA-29a/b/c was defined as one of the tumor-suppressive miRNAs in squamous cell carcinoma [53]. It is worth mentioning that miRNA-29a/b is involved in the Nrf2-desmocollin-2 axis, resulting in structural abnormalities of desmosomes in the pathogenesis of bullous diseases [53]. Increased levels of miRNA-223 were found in skin changes in toxic epidermal necrolysis, psoriatic plaques and skin lesions of hidradenitis suppurativa due to abundant inflammatory infiltrate, while lower miRNA-203 expression was also found in lichen planus, indicating its additional role in apoptosis and inflammation on disease progression [46,54,56,133]. Regarding anti-miRNA-203 implementation in the treatment, experimental studies revealed the positive effects in wound healing, reduced fibrosing and scar formation, and the topical nanodelivery via elastic liposomes might successfully modulate miRNAs levels in lesional skin and therefore could potentially be used in the treatment of psoriasis [43,45]. In research on the AD model, remarkable improvement of atopic inflammation was reached after the introduction of anti-miRNA-155-5p, suggesting great potential of this antagomiR in treating AD [43]. Comparably, a few studies managed to show significant improvement in AD models after administration of anti-inflammatory miRNAs mimics [64]. Hence, a favorable outcome was demonstrated for agomiR-10a-5p targeting hyaluronan synthase-3 that resulted in reduced IL-8, CCL5 and keratinocyte proliferation rate, or in the case of agomiR-124 transfection into keratinocytes that led to the decrease in IL-8, CCL5 and CCL8 levels via targeting p65 [64]. Comparably, several other studies described the silencing of IL-13-mediated inflammation via applying a miRNA-143 mimic or the suppression of TLR2-induced generation of IL-8, CCL20 and αTNF with a miRNA-146a mimic directly targeting TRAF6 and IRAK1 [64]. Additionally, the administration of agomiR-375-3p or agomiR-1294 showed inhibitory activity in the inflammatory response, ultimately causing barrier restoration in AD models [64]. In support of the hypothesis of miRNAs being involved in possible treatment mechanisms, the administration of belinostat, the pan-HDAC inhibitor, resulted in restoration of the epidermal barrier by upregulating miRNA-335 while promoting terminal keratinocyte differentiation [37]. For the role of TLR, another study demonstrated that the inhibition of TLR8 may play a protective role in AD [42]. In an experimental model, the authors showed that TLR8 induced the NF-κB/MyD88 signaling pathway with further production of IL-1, IL-6, IL-12, αTNF, γIFN, while its suppression resulted in reduced levels of chemokines, IgE and IL-4 [134]. The authors managed to modulate the TLR8 gene via vector miRNA derived from Salmonella enterica subsp. enterica serovar Typhimurium, suggesting the therapeutic potential of epigenetic alteration of TLR8 in severe chronic AD [42,134].

Moving onward, when evaluating novel anti-atopic treatment modalities, mesenchymal stem cell-derived extracellular vesicles (MSC-EVs) emerged as suitable option for conveying immunomodulatory properties to target tissues [135,136,137,138]. Therefore, one study on a murine model of AD demonstrated comparable efficacy of MSC-EVs to dupilumab. EVs exerted anti-inflammatory effects both in vivo and in vitro via miRNA-146a targeting AKT/NF-κB and MAPKs pathways, diminishing Th2-, Th1- and Th22-derived cytokines [135]. A former study detected the attenuation of AD symptoms, abated degranulated mast cells, reduced IL-5, CCL5 and IL-17, as well as macrophage inflammatory protein-2 (MIP2) in an AD mouse model, followed by the administration of human adipose tissue MSCs. Subsequent research identified the miRNA-122a/SOCS1 axis responsible for the regulation of Th1/Th2/Treg transcription factors: T-bet, GATA-3 and Foxp3, respectively [138].

Taking all of the above into account, exploring the therapeutic potential of miRNAs could rely on a new classification of medicines, known as ‘epidrugs’, that exert epi-modulatory and epi-regulatory functions [36,37,64].

## 5. Conclusions

Summarizing, our findings imply that miRNA-146a may contribute to the determination of a more suitable diagnostic biomarker of AD and as such may reflect its specific role in the development of AD, while miRNA-21, miRNA-29b, miRNA-143 and miRNA-223 may serve as severity biomarkers mirroring their influence on the disease progression.

This research advances the field by establishing the specific miRNA profile in atopic patients, emphasizing miRNA-146a, miRNA-21, miRNA-143, miRNA-223 and miRNA-29b, while providing a suitable base for future investigations. We believe this study makes a significant contribution to the scientific audience by addressing the need for more precise diagnostic strategies and the urgent identification of novel molecules that could serve as potential targets for future treatment options. Apart from dermatology, this multidisciplinary topic permeates allergology, immunology and molecular biology. Also, the results imply a novel role and possible future implementation in clinical practice guidelines and stepping further, in pharmaceutical science and drug production.

However, the study has a few potential limitations. In view of the number of patients, larger cohorts from multiple health centers encompassing all ages and ethnic groups would be more preferable to generalize results. Also, a more diverse patient cohort would be advisable, with special respect to a variety in AD forms (e.g., intrinsic vs. extrinsic AD) or subgrouping AD patients dependent on clinical phenotypes (e.g., classic flexural AD, ‘head-and-neck’ dermatitis, hand eczema and erythrodermic forms, etc.) and miRNA profile in creating novel pheno-endotypes of AD. Therefore, our results present preliminary signals that require confirmation through larger prospective cohorts and independent replication studies.

Future work should be aimed at exploring definitive biologic mechanisms by which miRNA-146a may be involved in eosinophil regulation and atopic inflammation in humans, or focusing on monitoring miRNA-146a, miRNA-21, miRNA-143, miRNA-223 and miRNA-29b during the administration of different therapeutic modalities, to establish their potential as treatment response biomarkers. Further research is needed to elucidate the contribution of combinatorial miRNAs in allergic inflammation, forming a major basis for creating precise, individually tailored treatment options.

## Figures and Tables

**Table 1:** Demographic and clinical information of the study population. AD—atopic dermatitis, AR—allergic rhinitis, As—asthma, FA—food allergy, SCORAD—SCORing Atopic Dermatitis, IgE—immunoglobulin E, Eos—eosinophils, y—year.

Variable	AD Patients	Healthy Controls	*p*-Value
Total (*n*)	50	50	/
Gender Male (*n*, (%)) Female (*n*, (%))	14 (28) 36 (72)	15 (30) 35 (70)	0.83
Age, years (average ± 95% CI)	32.62 (29.22–36.02)	36.4 (33.39–39.41)	0.09
Early onset in childhood (*n*, (%))	8 (16)	/	/
Disease duration (year) (average ± 95% CI)	7.22 (4.34–10.09)	/	/
<1 y (*n*, (%))	13 (26)	/	/
>1–5 y (*n*, (%))	20 (40)	/	/
>5–10 y (*n*, (%))	5 (10)	/	/
>10 y (*n*, (%))	12 (24)	/	/
SCORAD 0–24 Mild AD (*n*, (%)) 25–50 Moderate AD (*n*, (%)) 51–103 Severe AD (*n*, (%))	6 (12) 26 (52) 18 (36)	/ / /	/
Total IgE (IU/mL) (average ± 95% CI)	899.8 (544.45–1255.15)	69.76 (47.53–91.99)	*p* < 0.001
Eos (%) (average ± 95% CI)	4.73 (3.51–5.96)	2.68 (2.22–3.14)	*p =* 0.002
Comorbidities AR, yes (*n*, (%)) As, yes (*n*, (%)) FA, yes (*n*, (%))	27 (54) 7 (14) 5 (1)	/ / /	/

**Table 2:** Selected miRNAs in both groups (mean ± 95% CI). AD—atopic dermatitis, C—control group.

	**Ct (95% CI)**	***p*-Value**
miRNA-26a AD	29.11 (26.57, 31.65)	0.585
C	28.20 (25.96, 30.42)	
miRNA-21 AD	26.20 (24.0, 28.40)	0.573
C	27.04 (25.01, 29.07)	
miRNA-29b AD	32.94 (30.85, 35.03)	0.665
C	33.59 (31.44, 35.74)	
miRNA-223 AD	24.91 (22.71, 27.11)	0.717
C	24.15 (22.20, 26.10)	
miRNA-143 AD	34.19 (31.89, 36.49)	0.63
C	33.49 (31.64, 35.34)	
miRNA-203 AD	37.74 (35.64, 39.84)	0.739
C	37.78 (36.23, 39.34)	
miRNA-146a AD	33.09 (30.59, 35.58)	0.042
C	35.97 (33.13, 38.80)	

**Table 3:** Relative expression of selected miRNAs in both groups (mean ± 95% CI).

	**ΔCt (95% CI)**	***p*-Value**
miRNA-21 AD	−2.92 (−4.4, −1.43)	0.198
C	−1.15 (−3.45, 1.16)	
miRNA-29b AD	3.83 (2.06, 5.59)	0.31
C	5.4 (2.85, 7.95)	
miRNA-223 AD	−4.36 (−6.96, −1.77)	0.827
C	−3.97 (−6.5, −1.44)	
miRNA-143 AD	5.08 (2.53, 7.64)	0.899
C	5.3 (3.06,7.54)	
miRNA-203 AD	8.53 (5.92, 11.14)	0.436
C	9.85 (7.7, 12.01)	
miRNA-146a AD	3.97 (1.23, 6.72)	0.021
C	8.35 (5.81, 10.9)	

## Data Availability

The original contributions presented in this study are included in the article. Further inquiries can be directed to the corresponding author.
